# Assembly and comparative analysis of the complete mitogenome of *Rubus chingii* var. *suavissimus*, an exceptional berry plant possessing sweet leaves

**DOI:** 10.3389/fpls.2024.1504687

**Published:** 2024-12-23

**Authors:** Yujie Shi, Zhen Chen, Jingyong Jiang, Wenwu Wu, Yue Xin, Wei Zeng

**Affiliations:** ^1^ Zhejiang Provincial Key Laboratory of Plant Evolutionary Ecology and Conservation, College of Life Sciences, Taizhou University, Taizhou, China; ^2^ Institute of Horticulture, Taizhou Academy of Agricultural Sciences, Linhai, China; ^3^ State Key Laboratory of Subtropical Silviculture, College of Forestry and Biotechnology, Zhejiang A&F University, Hangzhou, China

**Keywords:** *Rubus chingii* var. *suavissimus*, mitochondrial genome, master circle structure, rearrangement, phylogenetic analysis

## Abstract

*Rubus chingii* var. *suavissimus* is a special berry plant of *Rubus* in the Rosaceae family. Its leaves contain high-sweetness, low-calorie, and non-toxic sweet ingredients, known as rubusoside. As a medicine and food biofunctional plant, it is a combination of “tea, sugar, and medicine.” In this study, the complete mitogenome of *R. chingii* var. *suavissimus* was successfully assembled and annotated based on PacBio HiFi sequencing technology. The mitogenome of *R. chingii* var. *suavissimus* was a typical master circle structure, spanning 432,483 bp and containing 34 unique protein-coding genes (PCGs), 20 tRNAs, and 3 rRNAs. The majority of these PCGs was subjected to purifying selection, and only one gene (*ccmB*) showed sign of positive selection. The mitogenome of *R. chingii* var. *suavissimus* contained a large number of repeats, and the homogeneous fragments transferring between plastid genome and mitogenome, with a total of 55 pairs of mitochondrial plastid sequences (MTPTs), and the total size was 56,913 bp. Comparative analysis showed that the non-coding region in the mitogenome of *R. chingii* var. *suavissimus* had undergone frequent rearrangements during evolution, but the coding region was still highly conserved. Furthermore, the maximum likelihood and Bayesian inference phylogenetic trees were reconstructed of 10 shared PCGs in 36 plant species. The topological structures of two phylogenetic trees were consistent with the APG IV classification system and had high support rates. In general, this study clarifies the mitogenome of *R. chingii* var. *suavissimus* and provides valuable insights into the genetic evolution of the Rosaceae family.

## Introduction

*Rubus* L. belongs to the Rosaceae family. According to statistics, more than 900 species have been identified around the world, mainly distributed in the temperate regions of the Northern Hemisphere (Moreno-Medina et al., 2018; Yu et al., 2022), and approximately 194 species have been found in China (Liu et al., 2021). Most of the *Rubus* plants are traditional medicinal plants in China and have high medicinal values. The fruits, roots, leaves, and other parts of some *Rubus* plants are used as medicines to dispel cold and dampness, stop bleeding and relieve pain, clear heat and toxic materials, promote blood circulation, remove blood stasis, strengthen the kidney, and astringent essence (Ansari et al., 2024; Kumar et al., 2024). Its fruits have high nutritional value and are often used for fresh food, or to make jams, juices, fruit wine, and vinegar (Foster et al., 2019). It is also often planted in gardens and used as an ornamental plant. Therefore, it is a type of plant that integrates medicinal, edible, and ornamental values, and has great development and utilization prospects.

*Rubus chingii* is one of the main representative species of *Rubus*, and it is the only *Rubus* plant recorded in the “China Pharmacopoeia.” In addition to the *R. chingii* var. *chingii*, this species also contains a special variety [*R. chingii* var. *suavissimus* (S.Lee) L.T. Lu], also known as Chinese sweet tea (Chou et al., 2009). It is distributed mostly on the hills and slopes of Guangxi Province in southern China. Although *R. chingii* var. *suavissimus* and *R. chingii* var. *chingii* belong to *R. chingii* species, there are significant differences in leaves between the two varieties. The leaves of sweet tea contain high-sweetness, low-calorific value, and safe sweet ingredients, which can be drunk as tea and used as medicinal materials (Abdel-Hamid et al., 2020). At present, sweet tea has been grown in China on a large scale, and its products have spread to Japan, the United States, and other countries. In recent years, extensive research has been carried out on the chemical composition (Koh et al., 2009; Liu et al., 2022), pharmacology (Chou et al., 2009; Jiao and Liu, 2020), and biosynthesis of sweet tea (Sun et al., 2018; Liu et al., 2023a). However, its genetic and molecular basis have not yet been clarified. Additionally, the species of *Rubus* are very diverse. Over the years, plant taxonomists from various countries have carried out a significant amount of research on their classification (Wang et al., 2016; Huang et al., 2023; Lu et al., 2024). However, different scholars have disagreement in the scope division of *Rubus* and the classification of subgenera. This has seriously hindered the development and utilization of this genus.

Mitochondria are semi-autonomous organelles with double membranes that are widely found in eukaryotes. They are also the place where cells undergo oxidative metabolism (Nunnari and Suomalainen, 2012). In addition, the genetic system of the mitogenome is predominantly maternally inherited, appears to be somewhat independent of the nuclear genome, and is generally more conservative in nature (Wang et al., 2024b). The evolution rate of plant mitogenome sequences is relatively slow, while the recombination rate of genomic structures is relatively fast (Bi et al., 2024b). This feature makes the mitogenome more suitable for resolving deep and large-scale phylogenetic relationships such as the rapid evolution of plastome groups and the early evolution of terrestrial plant systems (Tang et al., 2023). For example, the controversial evolutionary relationship between *Selaginella sinensis* and *S. sanguinolenta* in plastomes was effectively resolved (Tang et al., 2023). Therefore, the plant mitogenome is a valuable genetic resource for studying plant phylogeny and cellular processes. Due to the extreme complexity caused by frequent reorganizations and rearrangements, mitogenomes in most land plants are more difficult to be sequenced and assembled than many other eukaryotic lineages or chloroplast genomes (Liu et al., 2023b). In recent years, with the development of sequencing and assembly technology, some software dedicated to plant mitogenome assembly, such as Mitofiner (Allio et al., 2020), GSAT (He et al., 2023a), and PMAT (Bi et al., 2024a), have emerged, allowing the complete mitochondrial genome of plants to be successfully assembled, thus deepening our understanding of the inherent complex structure of plant mitochondrial DNA.

To date (as of 2 July 2024), 716 complete plant mitogenomes have been submitted in the NCBI database, including 65 species in the Rosaceae family and only one species in the genus *Rubus*, and most of the mitogenomes maintain the “Master circle” conformation (Wu et al., 2022). In recent years, the genomes of *Rubus* have been announced one after another (Wang et al., 2021; Yang et al., 2022; Wu et al., 2024), and the chloroplast genomes have also been extensively studied (Yu et al., 2022; Shi et al., 2024), but there are few reports of research on their mitogenomes (Sun et al., 2022), which has seriously hindered the in-depth understanding of biological characteristics at the cellular level. Thus, it is urgent to study the mitogenome of *Rubus* plants to further obtain information resources that will help future genetic evolution, phylogeny, and protection strategies.

This study conducted a comprehensive assembly and comparative analysis of the mitogenome of *R. chingii* var. *suavissimus*, including genomic characteristics, repetitive sequences, haplotype networks, codon usage bias, Ka/Ks ratio values, RNA editing sites prediction, and DNA sequence transfer. Multiple collinearity plots were used to compare six closely related species to determine whether genetic recombination and rearrangement occurred during the evolution of the mitogenomes among these species. Furthermore, based on shared PCGs from 36 species, the evolutionary relationship and genetic background of Rosaceae plants were further clarified. The deciphering of the mitogenome enriches the molecular markers and genetic resources for *Rubus* breeding and provides a basis for an in-depth understanding of the evolution from organelle genomes.

## Materials and methods

### Sample collection, DNA extraction, and sequencing

Fresh leaves of *R. chingii* var. *suavissimus* were collected at the Qingya raspberry planting base (120°1′56″ N, 29°9′7″ E), Yiwu, Zhejiang Province. The leaves were frozen in liquid nitrogen, and total DNA was extracted using a modified CTAB method (Abdel-Latif and Osman, 2017). The purity, integrity, and concentration of DNA were tested using a Qubit Fluorometer and agarose gel electrophoresis. The high-quality DNA samples that pass the test were sent to Novogen Biotechnology Co., Ltd. for second- and third-generation sequencing, respectively.

The second-generation sequencing was randomly broken into fragments by the ultrasonic cell crusher, and the entire library was prepared through steps such as end repair, adding ploy A, adding sequencing adapters, purification, and PCR amplification. The constructed library was subjected to PE sequencing by Illumina Novaseq platform. The raw reads obtained by sequencing was subjected to quality control through fastp v0.19.5 software (Chen et al., 2018) to obtain clean reads. Reads with the adapter and those with the ratio of N >10% were removed; the paired reads were removed when the single-read reads contain low-quality bases that exceed 20% of the length of the reads.

The third-generation sequencing used single-molecule real-time (SMRT) sequencing technology. The library was prepared by PacBio Binding kit, sheared DNA fragments with a main peak of approximately 15–18 kb were used, large fragments of DNA were enriched and purified using magnetic beads, and the fragmented DNA was end repaired. Then, stem-loop sequencing adapters were connected to both ends of the DNA fragments. The finally constructed library was sequenced through the PacBio Revio platform to generate raw data. Raw reads were interrupted from the adapter, and the adapter sequence were filtered out to obtain subreads, which were used to generate high-precision HiFi reads.

### Mitochondrial and chloroplast genome assembly and annotation

HiFi reads were used to assemble the mitogenome via PMAT v1.5.3 software (Bi et al., 2024a) with the “autoMito” model. The assembly parameters were set as follows: “-st HiFi -g 250M -tp mt -ml 40 -mi 90”. The genome size of *R. chingii* var. *suavissimus* was inferred from *k-mer* analysis of second-generation clean reads. The obtained initial assembly sequence was visualized through Bandage v0.8.1 software (Wick et al., 2015), and the chloroplast and nuclear contigs were manually removed. The HiFi reads were used to map the mitogenome of sweet tea to identify repetitive regions via Minimap2 v2.24 tool (Li, 2021) and ultimately generated a circular contig for *R. chingii* var. *suavissimus*. The chloroplast genome of *R. chingii* var. *suavissimus* was assembled using clean reads from second-generation sequencing by Getorganelle v1.7.7 software (Jin et al., 2020).

The mitogenomes of *R. chingii* var. *suavissimus* were annotated using the IPMGA (http://www.1kmpg.cn/ipmga/) and GeSeq (Tillich et al., 2017) tools, respectively, and the existing mitochondrial genomes of 65 Rosaceae plants were downloaded from the NCBI database as BLAST reference sequences in GeSeq. The chloroplast genome was also annotated via GeSeq tool. The tRNA and rRNA in the two genomes were annotated using tRNAscan-SE v2.0 tool and BLASTN software (Chen et al., 2015; Chan et al., 2021), respectively. The annotation information was visualized through Geneious v11.0.18 software (Kearse et al., 2012) to manually correct annotations. The mitogenome map of *R. chingii* var. *suavissimus* was visualized using the PMGmap tool (Zhang et al., 2024), and graphs of *cis-* and *trans-*splicing genes were drawn. Finally, the complete mitogenome of *R. chingii* var. *suavissimus* was uploaded to the GenBank database to obtain the accession number (PQ063984).

### Comparative analysis of the mitogenomes of Rosaceae

One species from each of 14 genera from the Rosaceae was taken and introduced into PhyloSuite v1.2.3 software (Zhang et al., 2020) for protein coding genes (PCGs) extraction with the mitogenome of *R. chingii* var. *suavissimus*. The extracted shared PCGs were subjected to multiple sequence alignment through the MAFFT v7.149b tool (Katoh and Standley, 2013). The sequences were trimmed after alignment using trimAl v1.2 software (Capella-Gutiérrez et al., 2009), and a maximum likelihood (ML) phylogenetic tree was constructed via IQ-TREE v2.2.0.3 software (Minh et al., 2020) with default parameters. In addition, haplotype sequences of shared PCGs were obtained through DnaSP v6.12.03 software (Rozas et al., 2003), and haplotype networks of 22 conserved protein coding sequences were constructed using the PopART v4.8.4 program (Leigh et al., 2015) with TCS Network model.

### Ka/Ks analysis

In order to evaluate the selection pressure of PCGs among *R. chingii* var. *suavissimus* and seven related species (*Argentina anserina*, *Fragaria ananassa*, *Geum urbanum*, *Prunus armeniaca*, *Rubus chingii*, *Rosa chinensis*, and *Spiraea chinensis*) and one outgroup (*Amborella trichopoda*), homogeneous PCGs were extracted through PhyloSuite software and alignment using the MAFFT tool. Then, the Ka and Ks ratios for each gene were determined using the KaKs_calculator v2.0 tool (Wang et al., 2010), and statistical analysis were performed through Excel 2021.

### Analysis of codon usage bias

The PhyloSuite software was used to conduct codon usage bias analysis on homologous PCGs in the mitogenomes of *R. chingii* var. *suavissimus* and seven related species and calculated the relative synonymous codon usage (RSCU) value and the number of codons corresponding to each amino acid. The Genepionee cloud platform (http://cloud.genepioneer.com:9929/#/) and ChiPlot online tool (https://www.chiplot.online/) were used to draw the results of RSCU and codon numbers, respectively. Moreover, in order to further determine the influencing factors of codon preference, we used the Genepionee cloud platform to determine the GC content and effective number of codon (ENC) of PCGs and plotted the corresponding ENC-plot. The ENC-plot analysis can explore the relationship between ENC and GC_3_ distributions. It was an effective method to visualize codon usage deviations in genetic data and was usually presented in the form of scatter points and standard curves.

### Analysis of repeat fragments

The repeat sequences include simple sequence repeats (SSRs), tandem repeats, and dispersed repeats. Among them, SSRs were identified using the MISA-web (Beier et al., 2017). The minimum number of repeats for SSR motifs of length 1–6 were set to 10, 5, 4, 3, 3, and 3, respectively. Tandem repeats were detected via the Tandem Repeat Finder (Benson, 1999), with default parameters. The REPuter (Kurtz et al., 2001) was used to identify dispersed repeats (including forward repeats, reverse repeats, palindromic repeats, and complementary repeats) with the Hamming distance of 3, and minimum repeat size and maximum repeat times were set to 30 bp and 5,000, respectively.

### RNA editing sites prediction

All PCGs in the mitogenome of *R. chingii* var. *suavissimus* were extracted using PhyloSuite tool, and all possible editing sites from C to U were predicted via the Deepred-Mt tool (Edera et al., 2021). Based on a multi-layer convolutional neural network, the threshold for the results was set at >0.9.

### Homologous sequences identification

Homogeneous sequences between chloroplast and mitogenome of *R. chingii* var. *suavissimus* were searched by BLAST software, with parameters set as identity >90% and e value < 1e^−5^. We analyzed homologous sequence regions and determined the sequence length, number, and gene type of mitochondrial plastid sequences (MTPTs). The sequences transfer map of chloroplast and mitogenome was drawn using the Advanced Circos program in TBtools v2.118 software (Chen et al., 2023). Furthermore, we also used BLAST software to homologous align the mitogenome of *R. chingii* var. *suavissimus* with those of seven related species and used the online tool Circoletto (https://bat.infspire.org/tools/circoletto/) to visualize.

### Mitochondrial genome rearrangement analysis

In order to explore whether the mitogenome of *R. chingii* var. *suavissimus* had undergone frequent rearrangements, we used *R. chingii* var. *suavissimus* and seven closely related mitogenomes to perform pairwise comparisons via MUMmer4 software (Marçais et al., 2018) with default parameters, and retained collinear regions >500 bp in length as blocks. Muli-collinearity plots were plotted using NGenomeSyn v1.39 software (He et al., 2023b).

### Phylogenetic analysis

To study the evolutionary relationship of *R. chingii* var. *suavissimus* in a broader context, we downloaded a series of different plant mitogenomes from the NCBI database, including angiosperm (Magnoliids, Monocots, Eudicots, *Nymphaea tetragona*, and *Amborella trichopoda*), gymnosperms, and bryophytes. These include four Magnoliids (*Chimonanthus praecox*, *Cinnamomum camphora*, *Liriodendron tulipifera*, and *Magnolia liliiflora*), eight Monocots (*Zantedeschia aethiopica*, *Cocos nucifera*, *Pandanus odorifer*, *Allium cepa*, *Asparagus officinalis*, *Zea mays*, *Oryza sativa*, and *Triticum aestivum*), 18 Eudicots (*Vitis vinifera*, *Citrullus lanatus*, *Arabidopsis thaliana*, *Populus alba*, *Rhododendron simsii*, *Chrysanthemum morifolium*, *Nicotiana tabacum*, *Salvia miltiorrhiza*, *Glycine max*, *P. armeniaca*, *Malus domestica*, *S. chinensis*, *R. chingii*, *R. chingii* var. *suavissimus*, *G. urbanum*, *R. chinensis*, *A. anserina*, and *F. ananassa*), and two gymnosperms (*Ginkgo biloba* and *Pinus taeda*). Accession numbers for mitogenomes of all species in the NCBI database can be found in **Supplementary Table S5**. At the same time, *Marchantia paleacea* and *Cyathodium cavernarum* were served as outgroups. The inclusion of these different populations made it possible to comprehensively evaluate the phylogenetic location of *R. chingii* var. *suavissimus*. The 10 PCGs common to all species were extracted through PhyloSuite software; MAFFT was used for multiple sequence alignment, and tirmAI tool was used for trimming. Subsequently, we used IQ-Tree software to rebuild the ML tree with 5,000 bootstrap replicates and also used MrBayes v3.2.7a software (Ronquist et al., 2012) to build the Bayesian inference (BI) tree. All parameter settings referred to previous research (Shi et al., 2024).

## Results

### Basic characteristics of *R. chingii* var. *suavissimus* mitogenome

**Figure 1:**
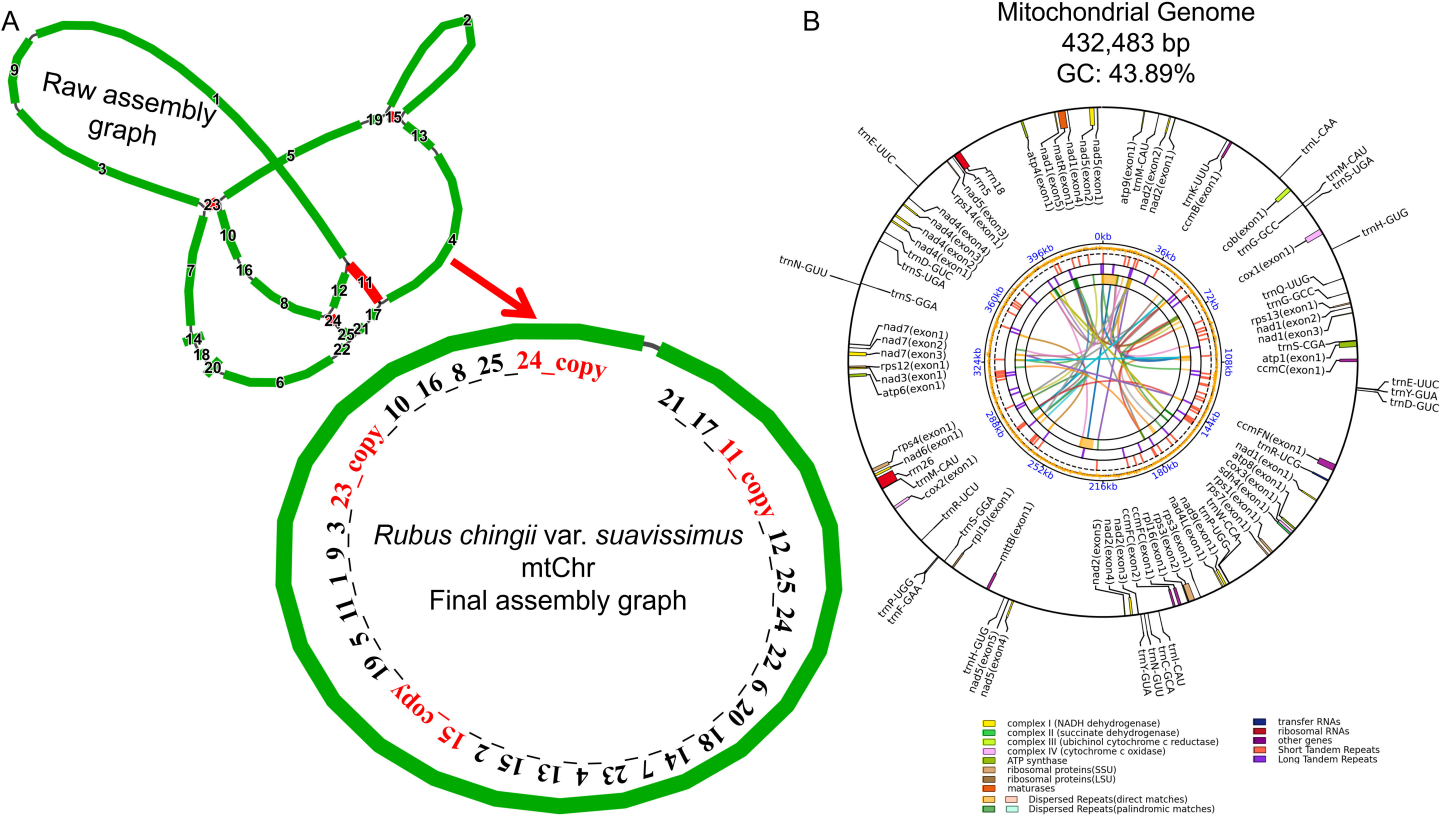
Assembly and annotation of *R. chingii* var. *suavissimus* mitochondrial genome. **(A)** The preliminary assembly graph of mitochondrial genome. Among them, contigs of different lengths are arranged with numbers, from long to short. Red contigs represent repeated fragments. The connection order of the mitochondrial sequence constituent circle is also presented in numerical order. **(B)** Mitochondrial genome annotation. Genes with different functions are given different colors. The colored parabolas in the center circle represent dispersed sequence repeats.

Based on HiFi reads sequencing data, we assembled the mitogenome of *R. chingii* var. *suavissimus*. Preliminary assembly result showed that the mitogenome of *R. chingii* var. *suavissimus* contained 20 contigs and four repetitive fragments (**Figure 1A**). These contigs were connected in order to form a circular mitogenome. The length of the mitogenome was 432,483 bp, and the GC content was 43.89%. The genome was annotated, and a total of 57 unique genes were obtained, including 34 protein-coding genes (PCGs), 20 transfer RNA (tRNA) genes, and three ribosomal RNA (rRNA) genes (**Figure 1B**). Among them, the core genes included five ATPase synthase genes (*atp1*/*4*/*6*/*8*/*9*), four cytochrome C genes (*ccmB*/*C*/*FC*/*FN*), one ubichinol cytochrome C reductase gene (*cob*), three cytochrome C oxidase genes (*cox1*/*2*/*3*), one mature enzyme gene (*matR*), one membrane transport protein gene (*matB*), and nine NADPH dehydrogenase genes (*nad1/2/3/4/4L/5/6/7/9*). Non-core genes included one succinate dehydrogenase gene (*sdh4*), two ribosomal large subunit genes (*rpl10/16*), and seven ribosomal small subunit genes (*rps1/3/4/7/12/13/14*) (**Supplementary Table S1**). In addition, *ccmFC*, *rps3*, and *trnR-UCG* contained only one intron, while *nad1/2/4/5/7* each contained multiple introns. The *ccmFC*, *nad4/7*, *rps3*, and *trnR-UCG* were identified as *cis-*splicing genes, and *nad1/2/5* were *trans-*splicing genes (**Supplementary Figure S1**).

### Comparative analysis of the mitogenome of *R. chingii* var. *suavissimus* and related Rosaceae species

In order to further explore the evolutionary characteristics of *R. chingii* var. *suavissimus* mitogenome, we compared it with 14 Rosaceae species. The GC content of the mitogenomes protein-coding regions from these 15 Rosaceae species ranged from 42.6% to 43.23%. Moreover, the GC_1_, GC_2_, and GC_3_ contents of these mitogenomes ranged from 47.79% to 48.29%, 42.35% to 42.87%, and 37.34% to 38.96%, respectively. Detailed values for each species can be found in **Supplementary Table S2**.

It was worth noting that although all 15 species belonged to the same family, there were large differences in the size of their mitogenomes, GC content, and gene numbers. According to our statistical analysis, the overall GC contents were distributed in the range of 43.3%–45.4%. *P. armeniaca* had the largest mitogenome size (510,346 bp), *F. ananassa* had the smallest mitogenome size (285,543 bp), with a difference of 224,803 bp, while the size of *R. chingii* var. *suavissimus* mitogenome was moderate. In terms of gene numbers, *P. armeniaca* and *R. chingii* var. *suavissimus* had the largest number of genes (57), while *G. urbanum*, *A. anserina*, and *F. ananassa* had the smallest number of genes (47). To further analyze these mitogenomes, we extracted PCGs from these species and identified 22 shared genes (*atp1/4/6/8/9*, *ccmB/C/FN/FC*, *nad3/4/5/6/7/9*, *rps3/13*, *cox1/2/3*, *matR*, and *cob*). Based on these shared genes, we constructed an ML phylogenetic tree (**Supplementary Figure S2**). The result showed that 15 species were divided into five groups, of which eight species had close genetic relationships with *R. chingii* var. *suavissimus* and were grouped into one clade.

**Figure 2:**
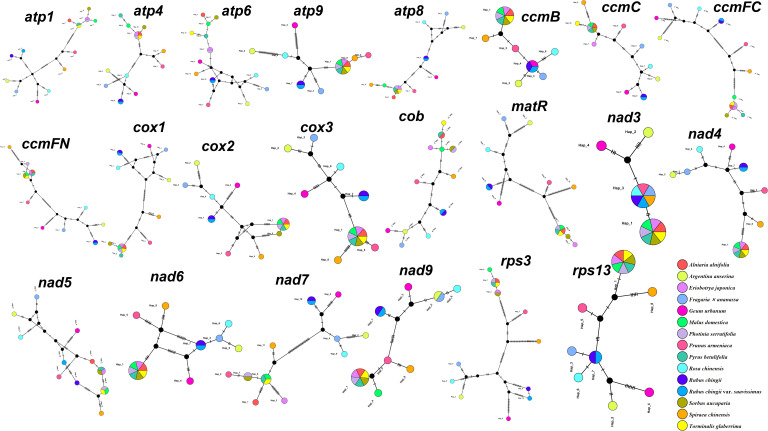
Haplotype network analysis of 22 shared protein-coding sequences from 15 Rosaceae mitogenomes. Different species are given different colors.

To further analyze the variation of shared genes between *R. chingii* var. *suavissimus* and related species, we constructed haplotype networks based on the sequences of 22 shared genes (**Figure 2**). It was worth noting that the haplotype composition of *S. chinensis*, *P. armeniaca*, *G. urbanum*, *R. chinensis*, *A. anserina*, and *F. ananassa* were typical and only shared one gene, while *R. chingii* and *R. chingii* var. *suavissimus* had the same composition in 19 genes. Furthermore, the remaining seven species (*A. alnifolia*, *P. betulifolia*, *M. domestica*, *P. serratifolia*, *T. glaberrima*, *E. japonica*, and *S. aucuparia*) shared at least 10 more genes with each other.

### Analysis of selection pressure of shared PCGs from Rosaceae species

**Figure 3:**
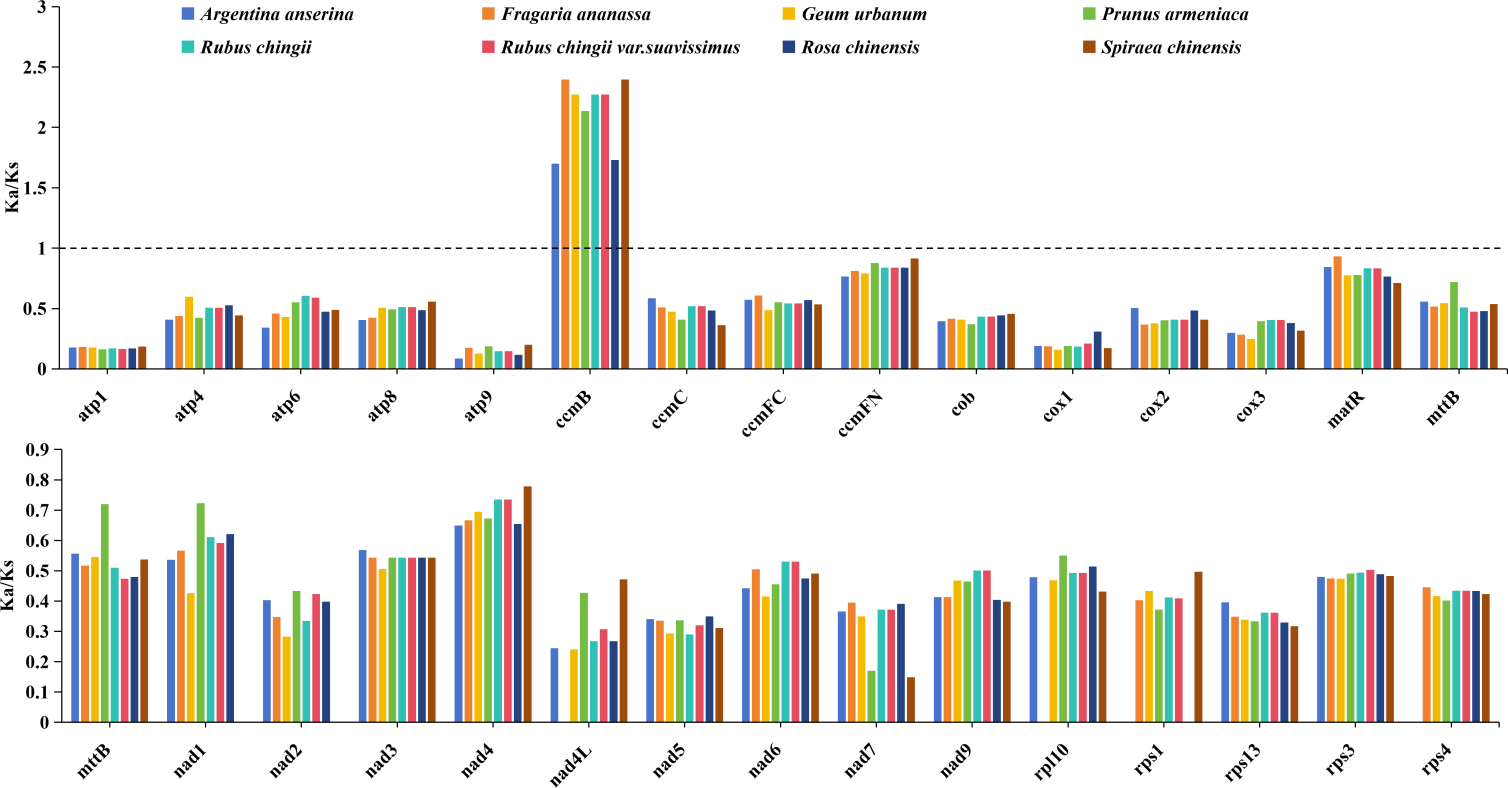
The Ka/Ks ratio values of 30 common PCGs in eight Rosaceae mitogenomes. Different species are given different colors.

To explore the impact of selection pressure on the evolution of *R. chingii* var. *suavissimus* mitogenome, we selected eight closely related species that grouped into the same clade as the *R. chingii* var. *suavissimus* and evaluated the ratio of non-synonymous substitutions (Ka) to synonymous substitutions (Ks) in the 30 shared PCGs (**Figure 3**). Our analysis showed that the trend of Ka/Ks ratio values for the 30 shared PCGs was similar among nine species, with most genes undergoing purifying selection, and Ka/Ks ratio values lower than 1. It was worth noting that only one gene (*ccmB*) had Ka/Ks values above 1 in all species, meaning that it had undergone potential positive selection in response to environmental adaptation.

### Analysis of codon usage bias in Rosaceae

**Figure 4:**
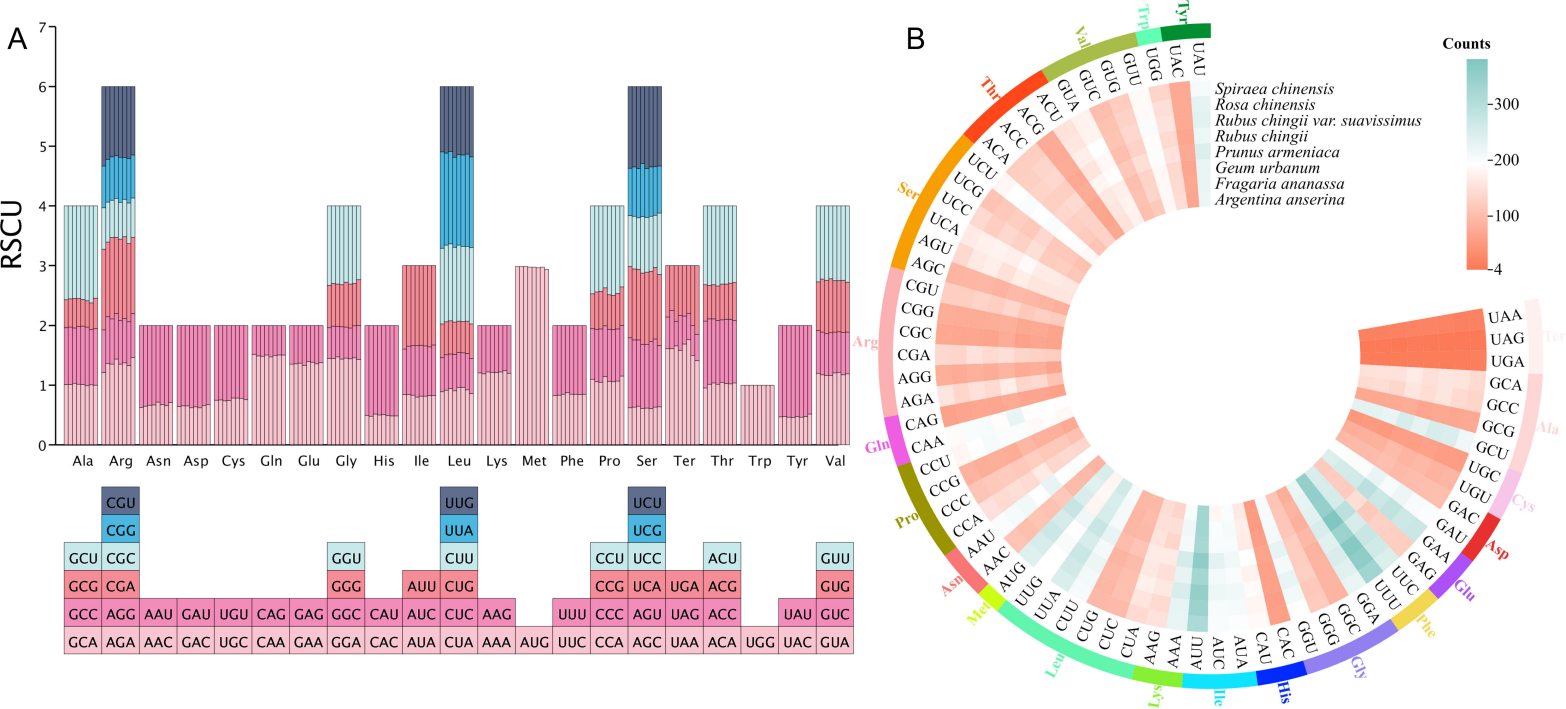
Codon usage bias analysis of mitogenomes from eight Rosaceae species. **(A)** Statistics of relative synonymous codon usage (RSCU) values for different species. Each bar represents a species, from left to right, namely, *A. anserina*, *F. ananassa*, *G. urbanum*, *P. armeniaca*, *R. chingii*, *R. chingii* var. *suavissimus*, *R. chinensis*, and *S. chinensis*. **(B)** Statistics on the total number of codons used for amino acids in protein-coding genes from different species.

Among the PCGs from the eight Rosaceae mitogenomes, we identified a total of 75,872 codons, distributed between 8,954 and 10,349 (**Figure 4B**). There was a total of 64 different types of these codons, encoding 20 amino acids and a stop codon, of which UUU was the most frequently used codon (335–381). Among these 20 amino acids, the trend in the number of codons used among eight species was similar. Leucine had the largest number of codons, with 977–1,094 codons (accounting for 10.66% of the total), followed by serine, with 802–934 codons (accounting for 8.99% of the total), while cysteine had the lowest codons among *A. anserina*, *F. ananassa*, *G. urbanum*, and *R. chinensis* (126–141), and tryptophan of remaining four species had the lowest number of codons (134–153). Additionally, we counted the RSCU values of 64 codons in different species (**Figure 4A**), and the results showed that the frequency of usage from 32 codons was higher than expectation, as reflected by the RSCU values >1. Among them, methionine (AUG) preference was the strongest, and the frequency of usage from 32 codons was lower than expectation (RSCU < 1), but the lowest frequently used codon varied slightly among different species. Interestingly, tryptophan (UGG) showed no codon usage bias (RSCU = 1). In terms of amino acids, except for tryptophan, the codon usage patterns of all amino acids showed preference, and tryptophan had only one codon similar to methionine, while most amino acids had at least two different codons, and arginine, serine, and leucine even had six different codons each.

In order to further explore the codon usage bias of *R. chingii* var. *suavissimus* mitogenome, we calculated the GC content at the first, second and third positions of 34 genes respectively, which were 38.12%–56.95%, 36.11%–54.55%, and 23.93%–58.08%, respectively (**Supplementary Table S3**). The average GC content at different positions was <50%, indicating that there was a bias for A/T and A/T ending in codons. Moreover, we calculated the ENC values of these genes, ranging from 37.3% to 56.89%, and the average was higher than 35%, indicating that the codon usage bias of *R. suavissimus* was relatively weak. The average GC content and ENC of the other seven related species were also <50% and >35%, respectively, which were consistent with the codon preference trend of *R. chingii* var. *suavissimus*. To further delve into the factors influencing the codon usage pattern of *R. chingii* var. *suavissimus*, we focused on studying these 34 PCGs and used their ENC and GC3 values to product ENC-Plot (**Supplementary Figure S3**). The results showed that most of the genes in the *R. chingii* var. *suavissimus* mitogenome were lower than the standard curve, and only one gene was above the curve. The result indicated that selection pressure played a very important role in shaping the codon preference of the *R. chingii* var. *suavissimus* mitogenome, and the remaining seven species also showed a similar trend.

### Repeat sequences detection and RNA editing sites prediction

**Figure 5:**
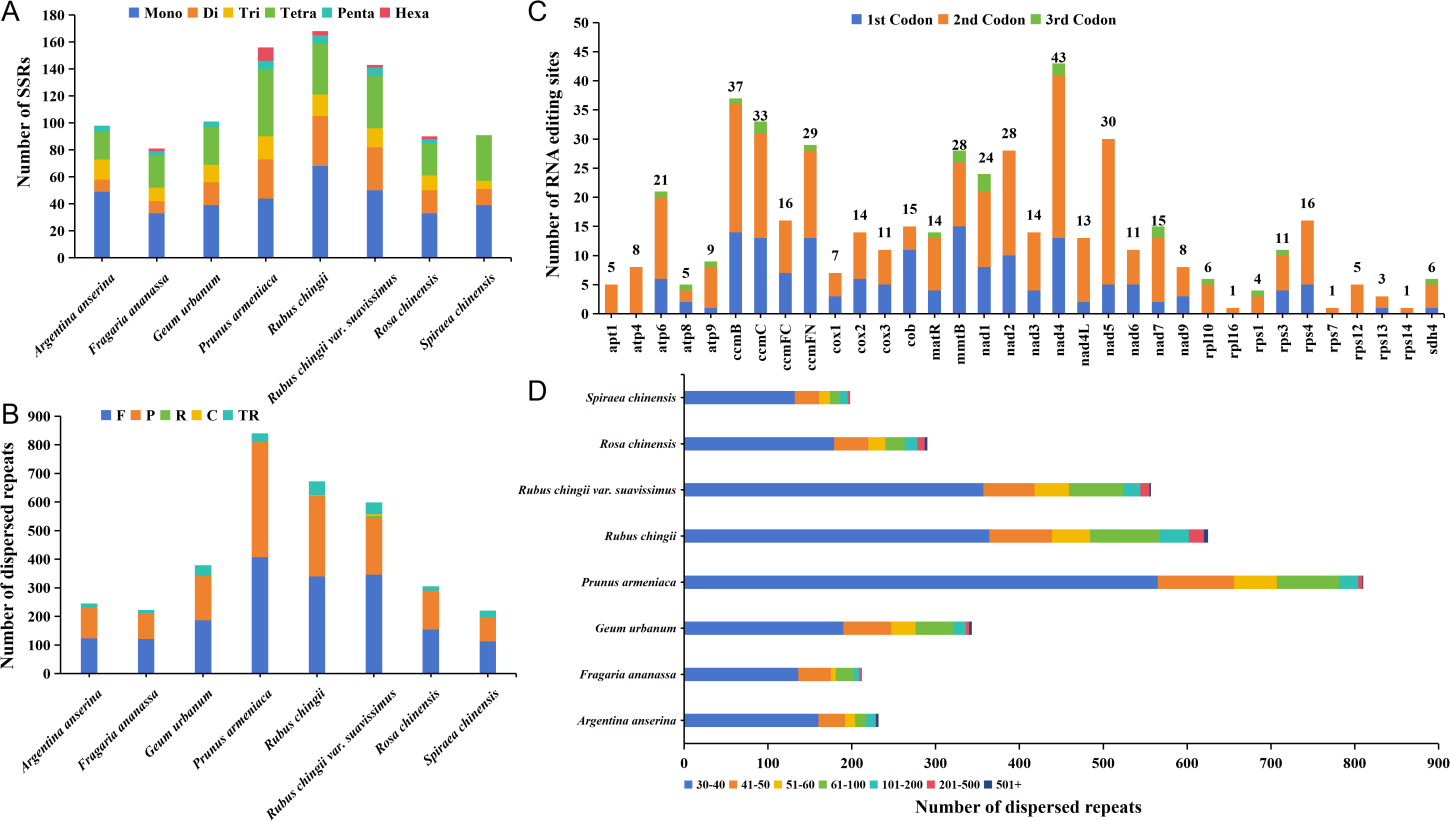
The different type sequence repeats analysis and RNA editing sites prediction. **(A)** Identification of SSR loci in eight Rosaceae mitogenomes. **(B)** Identification of dispersed sequence repeats from eight Rosaceae mitogenomes. **(C)** Prediction of RNA editing sites in the mitogenome of *R. chingii* var. *suavissimus*. **(D)** The length distribution of dispersed sequence repeats in eight Rosaceae mitogenomes. Mono, mononucleotide repeats; Di, dinucleotide repeats; Tri, trinucleotide repeats; Tetra, tetranucleotide repeats; Penta, pentanucleotide repeats; Hexa, hexanucleotide repeats; F, forward repeats; R, reverse repeats; P, palindromic repeats; C, complementary repeats; TR, tandem repeats.

The simple sequence repeats (SSRs) in the mitogenomes of eight Rosaceae were detected, and there was a total of 928 SSRs loci. Among them, the most SSRs loci were detected in the mitogenome of *R. chingii* (168), and the least number of SSRs loci was 81 in *F. ananassa* mitogenome (**Figure 5A**). Although the number of SSRs in the eight Rosaceae species varied greatly, the number of mononucleotide repeats dominated in all seven species, ranging from 33 to 68 (accounting for 0.35%–0.50% of all). *P. armeniaca* has the largest number of tetranucleotide repeats, and the number of hexanucleotide repeats was significantly higher than that of other seven species. Notably, *A. anserina* and *G. urbanum* lacked hexanucleotide repeats, *S. chinensis* lacked pentanucleotide and hexanucleotide repeats, and the rest of the species contained six repeat types. In addition, we detected tandem repeats and dispersed repeats (**Figure 5B**) in eight Rosaceae mitogenomes. The number of tandem repeats was distributed between 10 and 47, with large differences among individuals, and the trend of the number was consistent with SSRs loci. The number of dispersed repeats ranged from 198 to 810, and most species lacked complementary repeat and reverse repeat types. Only *R. chingii* and *R. chingii* var. *suavissimus* contained four repeat types, but the number of forward repeats (113–407) was the largest among all species, followed by palindromic repeats (85–402). Although the number and types of dispersed repeats varied among the eight species, the distribution trend of these sequence lengths was same, and they were mainly distributed between 30 bp and 40 bp, accounting for 55.39%–69.75% of the total (**Figure 5D**). Furthermore, the number of repeat sequences in *P. armeniaca* was much higher than that in the other seven species, which may be the main reason for the largest size of its mitogenome.

In this study, our goal was to predict possible C-to-U RNA editing sites in 34 different PCGs from the *R. chingii* var. *suavissimus* mitogenome to gain insight into gene expression. A total of 492 RNA editing sites were identified (**Figure 5C**), of which *nad4* had the most RNA editing sites (43), followed by *ccmB* (37), while most ribosomal proteins had generally fewer editing sites, and *rpl16*, *rps1*, and *rps14* even had only one editing site. It was worth noting that the second codon had the largest number of editing sites, with 308 (~62.6% of the total), while the number of editing sites for the third codon (21 sites, ~4.27% of the total) was much lower than the number of editing sites for the first and second codons. These RNA editing events often led to amino acid changes, most of which involved the conversion of hydrophobic amino acids, which will help improve protein stability.

### Intracellular genes transfer from chloroplast genome to mitogenome of *R. chingii* var. *suavissimus*

**Figure 6:**
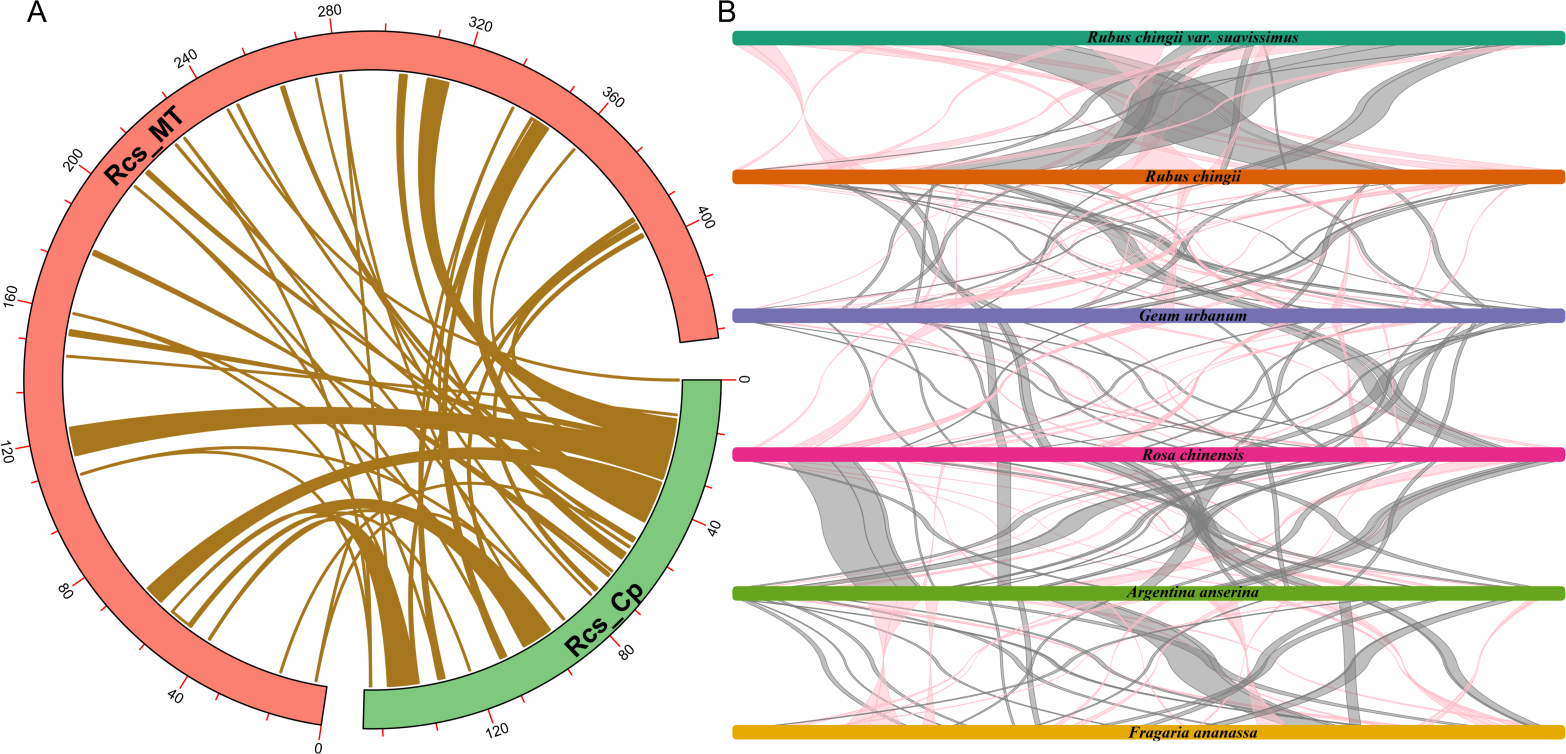
Homologous sequences analysis. **(A)** Homologue fragments between the chloroplast and mitochondrial genomes of *R. chingii* var. *suavissimus*. The red arc represents the mitochondrial genome. The green arc represents the chloroplast genome. The khaki lines represent the MTPTs. **(B)** Multicollinearity between *R. chingii* var. *suavissimus* and seven related species. Different colored bars represent the mitochondrial genome, the red line areas indicate the reversal occurred, and the gray areas indicate good similarity. Blocks with a length >500 bp are reserved.

The gene transfers between organelles and nuclear were very frequent during the evolution of higher plants. These gene transfers mainly included the transfer of nuclear genomes to mitogenomes, the plastid genomes to mitogenomes, the mitogenomes to nuclear genomes, and the plastid genomes to nuclear genomes (Martin et al., 1998; Zhao et al., 2019). Through homologous sequence alignment between the mitochondrial and chloroplast genomes of *R. chingii* var. *suavissimus* (**Figure 6A**), a total of 55 pairs of MTPTs were found, with sequence lengths ranged from 30 bp to 7,618 bp, containing a total of 56,913 bp, accounting for 13.16% of the mitochondrial genome (**Supplementary Table S4**). Among these similar fragments, MTPTs-1 and MTPTs-2 were the largest, with a length of 7,618 bp. We continued to annotate these 55 pairs of similar sequences and identified 27 complete genes, including 14 PCGs (*psbC*, *psbM*, *psbZ*, *ndhB*, *petG*, *petN*, *atpH*, *atpI*, *rps2*, *rps7*, *rps12*, *rps14*, *rpoB*, and *ycf3*) and 13 tRNA genes (*trnD-GUC*, *trnE-UUC*, *trnG-GCC*, *trnH-GUG*, *trnI-CAU*, *trnL-CAA*, *trnM-CAU*, *trnN-GUU*, *trnP-UGG*, *trnS-GCU*, *trnS-UGA*, *trnW-CCA*, and *trnY-GUA*). It contained a large number of genes related to photosynthesis and self-repair, which may be related to the adaptive evolution of the organelle genome of *R. chingii* var. *suavissimus*.

### Multiple collinearity analysis among *R. chingii* var. *suavissimus* and five related species

Compared to chloroplast genomes, plant mitogenomes undergo more frequent rearrangement events (Palmer and Herbon, 1988). To further evaluate the relationship between *R. chingii* var. *suavissimus* mitogenome and five Rosaceae mitogenomes, we performed multiple collinearity analysis, retaining conservative collinearity blocks more than 500 bp in length to show similarity and sequence arrangement (**Figure 6B**; **Supplementary Figure S4**). The results showed that *R. chingii* var. *suavissimus* and five other Rosaceae species had many identical collinear blocks, among which there were a large number of longer collinear blocks with *R. chingii* (824 bp–55,215 bp, accounting for approximately 97.43% of *R. chingii* var. *suavissimus* mitogenome), while the collinear blocks with other species were shorter in length, indicating that species with closer relatives had the opportunity to retain longer collinear blocks. In addition, the collinear blocks among the mitogenomes contained a large number of conserved genes, but the overall arrangement order showed inconsistency, indicating that six species had experienced frequent gene rearrangements, resulting in shorter collinear blocks, while the protein coding regions remain highly conserved. The findings of these results highlighted the extremely unstable structure of the plant mitochondrial genome, but its protein coding regions were quite conserved.

### Phylogenetic analysis of multiple species

**Figure 7:**
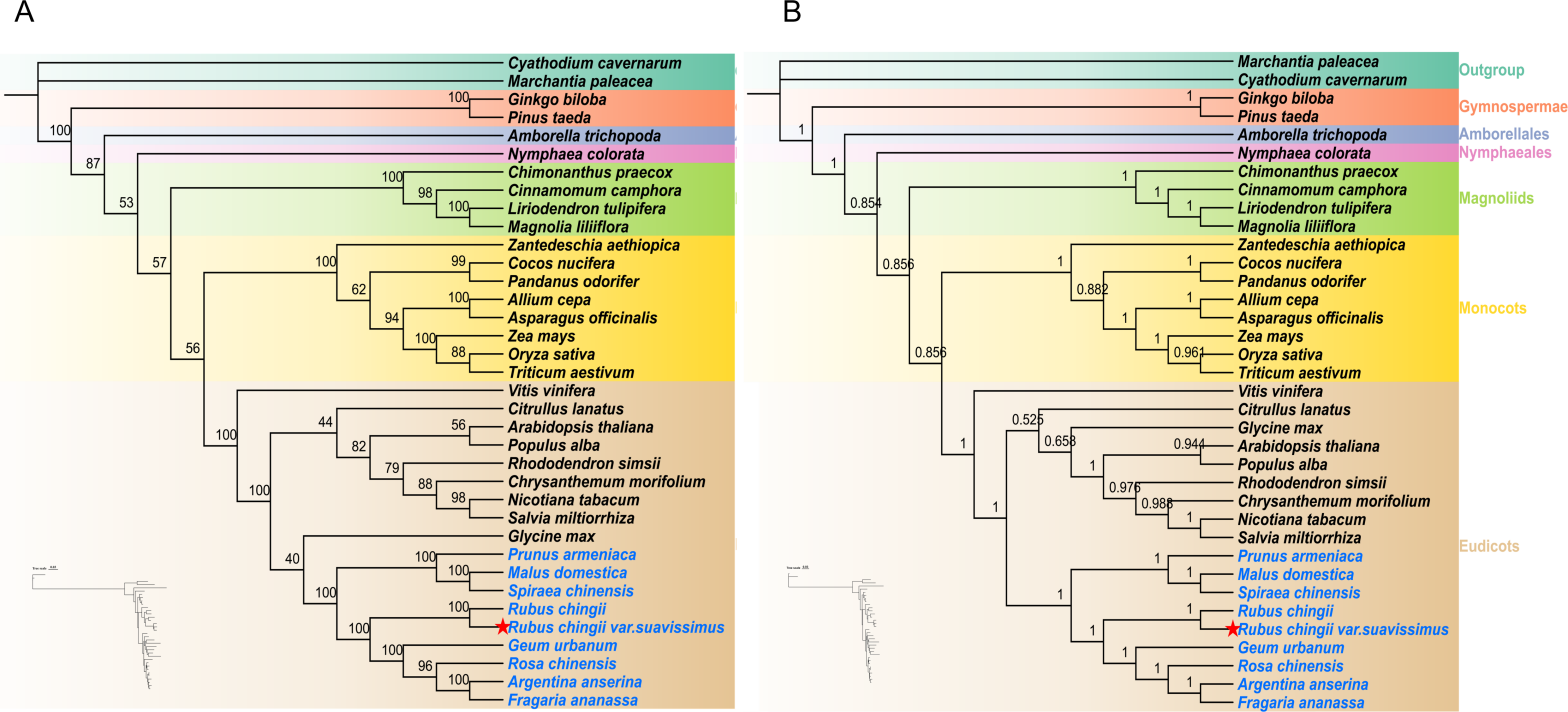
The maximum likelihood (ML) phylogenetic tree **(A)** and Bayesian inference (BI) phylogenetic tree **(B)** were constructed based on 10 shared PCGs from the mitogenomes of 36 species. The support rates of the evolutionary trees are shown on the branches.

To explore the phylogenetic location of *R. chingii* var. *suavissimus*, we analyzed 36 complete mitogenomes, including angiosperms (Monocots, Eudicots, Magnoliids, *N. colorata*, and *A. trichopoda*) and gymnosperms, with bryophytes as outgroups. The ML and BI phylogenetic trees were reconstructed based on 10 shared PCGs (*atp1/6/9*, *ccmB*, *cox2/3*, *cob*, and *nad3/5/6*), and each branch node showed a high support rate (**Figures 7A, B**). *R. chingii* var. *suavissimus* had the closest kinship with *R. chingii* and then formed a small clade with *G. urbanum*, *R. chinensis*, *A. anserina*, and *F. ananassa*. The topological structures of ML and BI trees were generally consistent, and the phylogenetic structure shown was consistent with the latest classification of APG IV. The only difference lied in the phylogenetic relationship between Rosaceae and other dicotyledonous plants. In the ML tree, *G. max* had a close genetic relationship with Rosaceae species. Rosaceae and *G. max* were first grouped into a group, then formed a big branch with other dicotyledonous plants, but the support rate was low. In the BI tree, Rosaceae plants were monophyletic taxa. After Rosaceae grouped into a clade alone, they formed a large branch with other dicotyledonous plants and received a high support rate. Therefore, the BI tree based on the mitochondrial genome may better demonstrate the phylogenetic relationships among species.

## Discussion

Most mitogenomes in higher plants are presented in complex circles or lines and are likely to be complexes of multiple configurations, mainly caused by frequent recombination mediated by a large number of repetitive sequences (Kubo and Newton, 2008; Smith and Keeling, 2015). Generally, forward and inverted repeat sequences mediated recombination produces large/small subring molecules and isomers, respectively (Wang et al., 2024a). A large number of studies have clarified the diversity of mitogenome conformations in different plants. For example, the cucumber mitogenome consists of a large loop of 1.6 Mb and two small loops of 84 kb and 45 kb in size, respectively (Alverson et al., 2011), while the mitogenome of *Mentha spicata* contains a long linear structure and two smaller circles (Jiang et al., 2023). The mitogenome of *Silene conica* even contains 128 circular chromosomes, each of which varies in size (Sloan et al., 2012). In this study, we sequenced, assembled, and reported for the first time the complete mitogenome of *R. chingii* var. *suavissimus*, a special variety of *R. chingii*. Its mitogenome presented a typical master circle structure with a total length of 432,483 bp. In the mitogenome of sweet tea, a total of 57 functional genes were identified, including 34 unique PCGs, 20 tRNA, and 3 rRNA genes. Remarkably, the GC content plays an important role in determining amino acid composition during the evolution of higher plants (Du et al., 2018). The GC content in the mitogenome sequence of sweet tea is approximately 43.89%, which is similar to the GC content observed in the mitogenomes of 14 species from the same family (43.3%–45.4%). The same phenomenon was also observed in Apiales species (Wang et al., 2024a), but there were huge differences in GC content among some seed plants (*Elymus sibiricus*, *Saccharum officinarum*, and *Oryza sativa*) (Xiong et al., 2022). The Ka/Ks ratio value has a complex relationship with the biological functions of PCGs and reflects gene selection pressure, thereby further understanding the evolutionary dynamics of PCGs among related species (Wang et al., 2010). It provides a perspective and method for in-depth understanding of biological adaptability. When we compared 30 PCGs from sweet tea and seven related species, the majority of genes was purifying selection and only one gene (*ccmB*) showed signs of positive selection. The high Ka/Ks ratio value of the cytochrome c biogenic gene *ccmB* suggests its important role in evolutionary species and may be related to environmental adaptation.

Codons are the basic carriers for accurate identification and transmission of genetic information in nature (Li et al., 2023). Codons encoding the same amino acid are called synonymous codon. Codon usage bias plays an important role in nature during the process of inheritance and variation (Quax et al., 2015). During protein translation, the frequency of use of one synonymous codon corresponding to each amino acid is higher than that of other synonymous codons, which is called codon usage bias (Romero et al., 2000). In this study, we used 34 different PCGs to assess GC contents and RSCU values at different locations in the sweet tea mitogenome. The results showed that the third position of the mitochondrial gene sequence of sweet tea tended to the A/T and the codon ending in A/T. This finding is consistent with previous research on the mitogenomes of *Mangifera* species (Niu et al., 2022) and *Angelica sinensis* (Wang et al., 2024a), indicating a certain degree of similarity in mitochondrial codon usage preferences among different species. ENC-plot showed that codon use in the mitochondrial genes of sweet tea and seven other related species was more influenced by natural selection, which is consistent with previous findings in other plants (Tang et al., 2020; Wang et al., 2024a).

In a comprehensive comparative analysis between sweet tea and five related species, it was found that the mitogenomes of sweet tea and its corresponding species experienced frequent gene rearrangements, and a large number of functional genes were found in homologous collinear blocks, which contributed to the evolution and expression regulation of the genome. In addition, repetitive sequences in plant mitochondrial genomes play an important role in promoting species evolution and can mediate gene recombination, resulting in an increase in the size of the mitogenomes (Bi et al., 2022; Zeng et al., 2024). In the mitogenome of sweet tea, a total of 143 SSRs, 42 tandem repeats, and 557 dispersed repeats were identified. These repetitive fragments help resolve the master circle conformation in the mitogenome of sweet tea. We also compared the repeat sequences of seven other related species and found that *P. armeniaca* had the most repeat sequences and was significantly more than other species, which might be the reason why it had the largest mitogenome. Numerous studies have found that the dynamic nature of homologous fragments extends from chloroplasts to mitochondrial organelles, highlighting the genetic architecture of interconnection and flow of these components (Wu et al., 2022). We successfully identified 55 MTPTs, ranging in length from 30 bp to 7,618 bp, distributed between sweet tea chloroplasts and mitochondrial organelles. The total length of these homologous fragments is 56,913 bp, accounting for 13.16% of the sweet tea mitogenome, which is significantly larger than that of *Angelica biserrata* (3.46%), *Panax notoginseng* (3.11%), willow (3.28%), and other plants (Han et al., 2022; Yang et al., 2023; Wang et al., 2024a), and similar to *R. chingii* in the family Rosaceae (16.34%) (Sun et al., 2022). Further annotation of these fragments revealed 27 complete genes, including 14 PCGs (*psbC*, *psbM*, *psbZ*, *ndhB*, *petG*, *petN*, *atpH*, *atpI*, *rps2*, *rps7*, *rps12*, *rps14*, *rpoB*, and *ycf3*) and 13 tRNA genes (*trnD-GUC*, *trnE-UUC*, *trnG-GCC*, *trnH-GUG*, *trnI-CAU*, *trnL-CAA*, *trnM-CAU*, *trnN-GUU*, *trnP-UGG*, *trnS-GCU*, *trnS-UGA*, *trnW-CCA*, and *trnY-GUA*). It is worth noting that among these genes, 14 PCGs are involved in photosynthesis and self-repair in the sweet tea chloroplast genome. In addition, these 13 tRNA genes may have changed or become pseudogenes in the mitogenome. In summary, there is intracellular gene transfer (IGT) between mitochondria and chloroplast organelles, a common phenomenon in higher plants (Bergthorsson et al., 2003), but conflicts with the assumption that most transferred genes are tRNA genes (Liu et al., 2023b).

There is a large number of RNA editing phenomena in the mitogenomes of higher plants, which is one of the necessary steps in plant mitochondrial gene expression (Kubo and Newton, 2008). Current research shows that the type with the most RNA editing sites are C-to-U, and U-to-C are also present in some species (Grewe et al., 2011). RNA editing occurs most frequently in the protein coding region and also occurs in some tRNAs, introns, and non-transcribed regions (Kan et al., 2020). RNA editing tends to occur at the first and second positions of the codon, and most edits change the amino acid type, usually converting hydrophilic amino acids to hydrophobic amino acids to facilitate protein folding to produce the desired function (Jackson et al., 2012). RNA editing also often acts on the creation of start and stop codons. Overall, protein homology increased among different species after RNA editing (Gray, 2009). In the mitogenome of sweet tea, a total of 492 RNA editing sites were identified, of which *nad4* showed the highest RNA editing efficiency, consistent with the results in *Cinnamomum camphora* (Han et al., 2024). This may have an impact on the stability of the mitochondrial respiratory chain complex enzyme. Furthermore, ACG-to-AUG editing occurred in *nad4L*, causing the gene to produce the correct start codon. In order to further clarify the phylogenetic relationship between Rosaceae plants and other angiosperms and gymnosperms, we used 32 angiosperms, two gymnosperms, and two bryophytes as outgroups to construct ML and BI phylogenetic trees. The results reflected that the classification of angiosperms in ML and BI were consistent with the APG IV classification system, and the BI phylogenetic tree had higher recognition for Rosaceae plants. However, a more comprehensive understanding of the phylogenetic relationships of *Rubus* species requires more extensive analysis with more molecular data on *Rubus* species.

## Conclusion

In this study, we assembled and annotated the mitogenome of *R. chingii* var. *suavissimus* based on HiFi reads and analyzed the detailed characteristics of the genome, including GC content, haplotype network, codon usage bias, repetitive sequences, RNA editing events, phylogenetic relationships, sequence migration, and rearrangement. The mitogenome of sweet tea is a single master circle conformation with a length of 432,483 bp, containing 34 PCGs, 20 tRNA, and 3 rRNA genes, and GC content is 43.89%. A comparative analysis showed that there were abundant repetitive sequences, frequent IGT, and rearrangement events in the mitogenome of sweet tea, which may be responsible for increasing the diversity and complexity of the genome. The mitogenome of sweet tea provides an important basis for subsequent research on genomic breeding, seed resource innovation, and systematic evolution of *Rubus*.

## Data Availability

The datasets presented in this study can be found in online repositories. The names of the repository/repositories and accession number(s) can be found in the article/**Supplementary Material**.
