# Supplementary material for: Assembly and comparative analysis of the complete mitogenome of Rubus chingii var. suavissimus, an exceptional berry plant possessing sweet leaves
Source: Front Plant Sci. 2024 Dec 23;15:1504687. doi: 10.3389/fpls.2024.1504687 (PMC11701164; doi:10.3389/fpls.2024.1504687)
Supplement: Supplementary file 1 [file Image1.pdf]

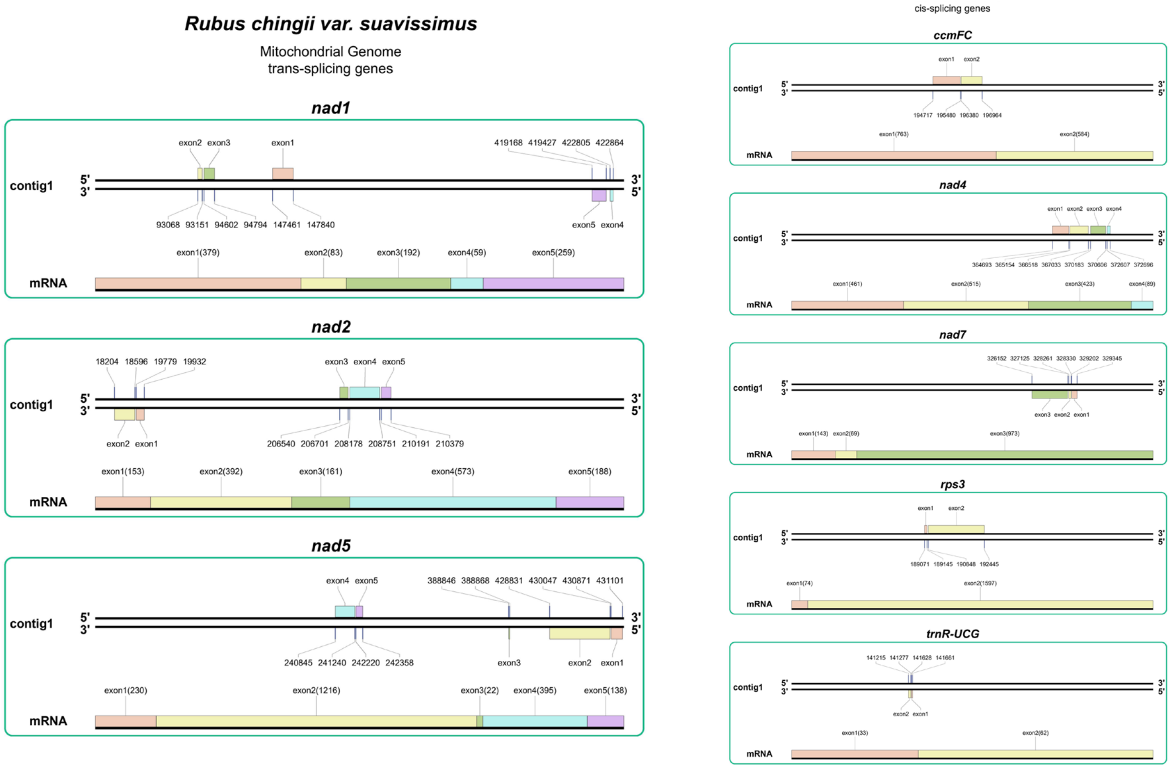

Figure S1 Identification of *cis*- and *trans*-splicing genes in the mitochondrial genome of *R. suavissimus*

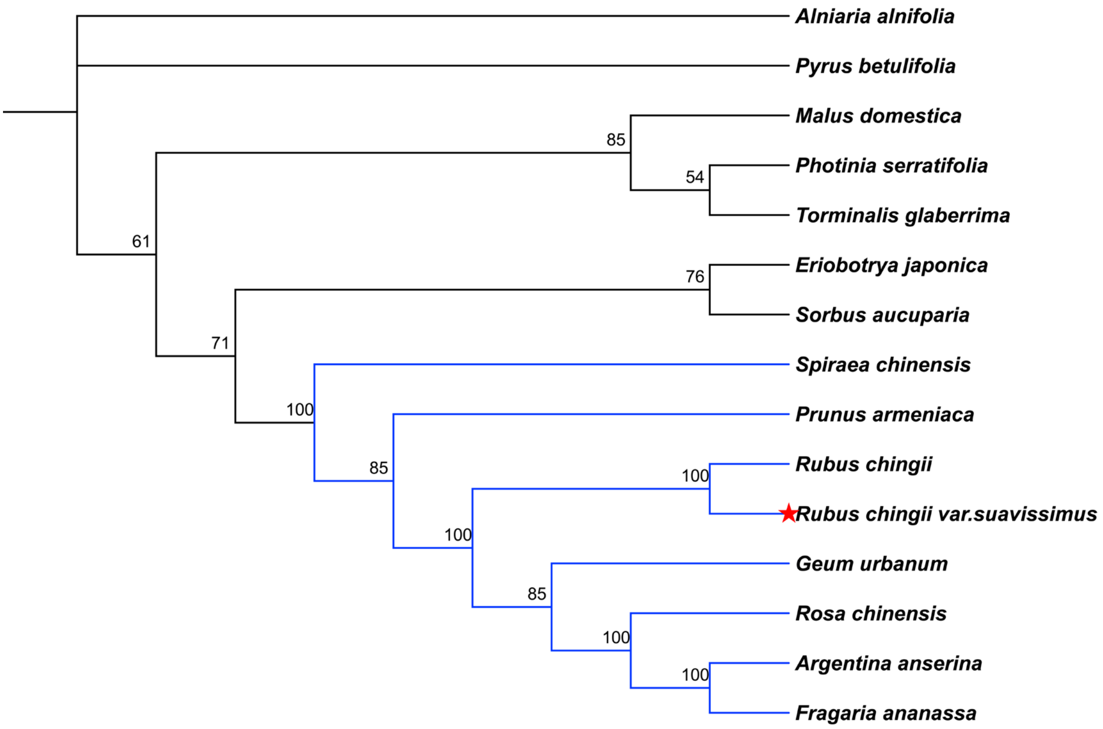

Figure S2 The ML phylogenetic tree was constructed based on 22 shared PCGs from 15 different genera of Rosaceae. The mitochondrial genome of *R. suavissimus* assembled in this study is highlighted by a red five-pointed star, and the species more closely related to it are represented by blue.

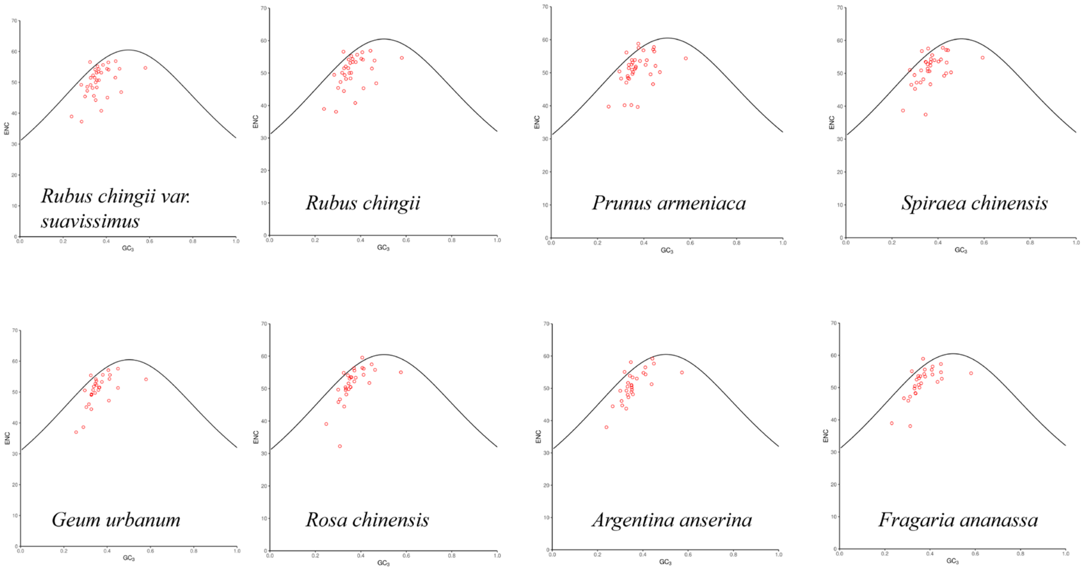

Figure S3 The ENC-plot of PCGs from 8 Rosaceae species. The black curves are the standard curves, the formula is ENC = 2 + GC_3 + 29/ [GC_3^2 + (1 - GC_3)^2], and each red dot represents a gene.

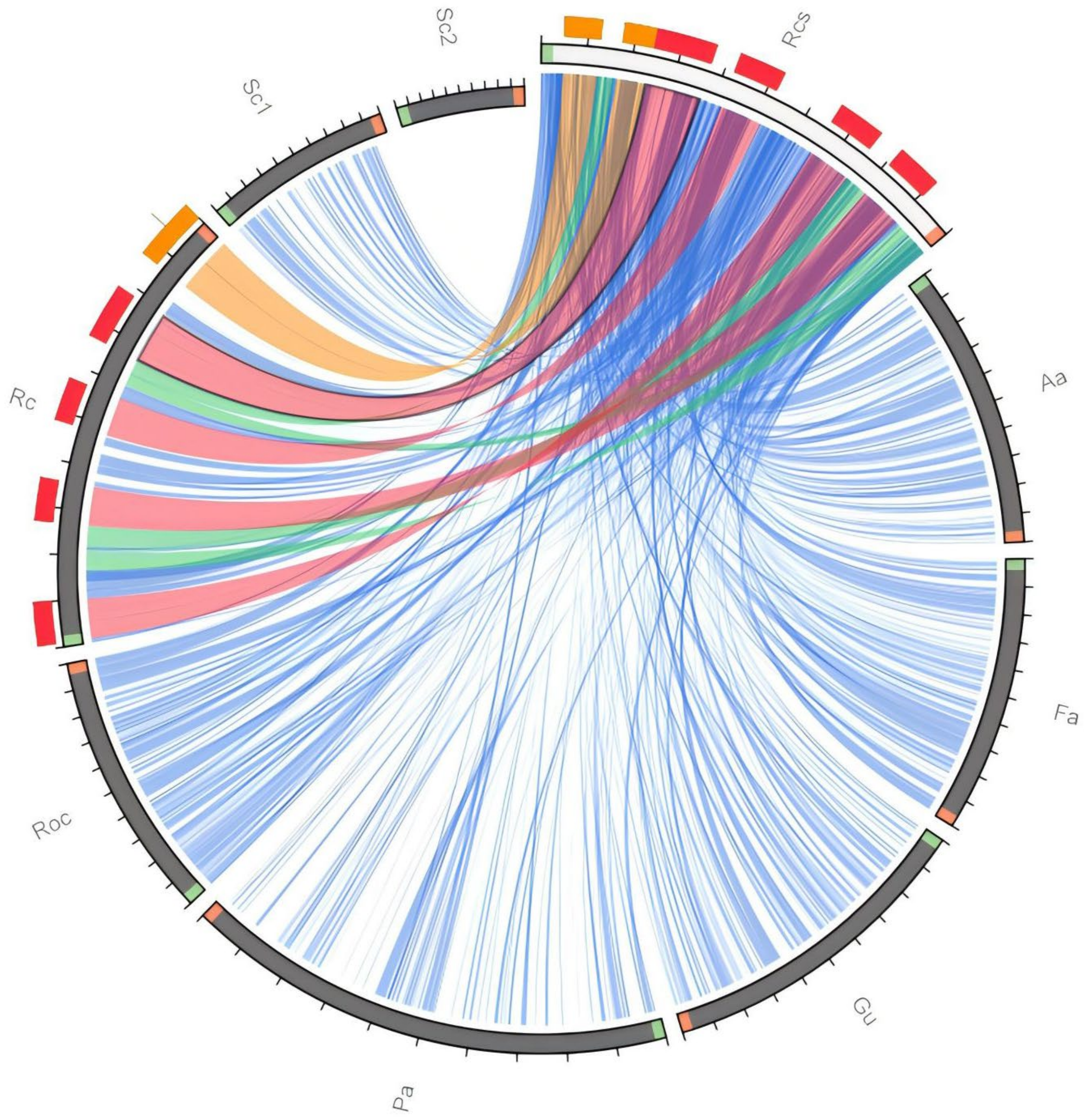

Figure S4 Collinearity analysis between *R. suavissimus* and 7 related species of Rosaceae. The mitogenomes of different species are represented by gray arcs, blue lines in the circle represent homologous fragments between *R. suavissimus* and 7 species respectively, while lines in other colors represent collinearity between sweet tea and the species with large segments, with the highest similarity. Rcs: *Rubus chingii* var. *suavissimus*, Rc: *Rubus chingii*, Gu: *Geum urbanum*, Roc: *Rosa chinensis*, Aa: *Argentina anserina*, Fa: *Fragaria ananassa*, Pa: *Prunus armeniaca*, Sc1and Sc2: *Spiraea chinensis*.
